# Mixed convective flow of CNTs nanofluid subject to varying viscosity and reactions

**DOI:** 10.1038/s41598-021-02228-9

**Published:** 2021-11-24

**Authors:** Zakir Hussain, Tasawar Hayat, Ahmed Alsaedi, Muhammad Shoaib Anwar

**Affiliations:** 1 Department of Mathematics, University of Baltistan, Skardu, 16100 Pakistan; 2 Department of Mathematics, Quaid-I-Azam University 45320, Islamabad, 44000 Pakistan; 3 Nonlinear Analysis and Applied Mathematics (NAAM) Research Group, Department of Mathematics, Faculty of Science, King Abdulaziz University, P.O. Box 80257, Jeddah, 21589 Saudi Arabia; 4 Department of Mathematics, University of Jhang, Gojra Road, Jhang, 35200 Pakistan

**Keywords:** Carbon nanotubes and fullerenes, Nanoparticles

## Abstract

The addressed work explains SWCNTs (Single walled carbon nanotubnes) and MWCNTs (Multi walled carbon nanotubnes) nanofluid flow under the influences of temperature dependent viscosity and mixed convection. Comparative study of SWCNTs and MWCNTs suspended in base liquid is presented. Further heat and mass transfer are addressed for nanofluid effected by radiation, heat generation/absorption and diffusion species. Mathematical development of problem is taken in cylindrical coordinates. System of highly nonlinear differential equations are constructed via appropriate transformations. The system of equations are tackled numerically by bvp4c MATLAB solver. The findings of the study show that larger volume fraction $\left( \phi \right)$ contributes to enhance the nanoliquid flow. The velocity by submerging MWCNTs is noted higher than SWCNTs. Furthermore, the relationship between the viscosity variable $\left( \theta _{r}\right)$ and the temperature is such that the temperature near the surface decreases with increase in $\left( \theta _{r}\right)$, while at the same time the temperature away from the surface increases. Subsequently, higher temperature is observed in SWCNTs-liquid compared to the MWCNTs-liquid to the similar values of $\left( \theta _{r}\right)$. Further, heat transfer is an increasing function of varying viscosity variable $\left( \theta _{r}\right)$.

## Introduction

The challenges and demands of advanced industries have been attracted the attention of researchers. The solar collectors used in devices are mostly affected by poor conductivity and low heat up capability of ordinary liquids. Choi^1^ was the first one to take lead to enhance thermal conductivity in this way by mixing nano-sized particles in ordinary liquids. Nanoliquids are composed by suspension of chemical stable nano-scaled materials namely oxides, metals and carbides etc in base liquids. These novel fluids are termed as nanoliquids which enhance thermal characteristics of base liquids (engine oil, water, glycol etc). Ordinary liquids namely engine oil, water, glycol etc having low conductivity. Suspension of nanoparticles in low thermal conductivity liquids advance the thermal conductivity and hence accelerates the performance of industrial liquids. Entropy analysis on nanoliquid flow is discussed by Meshal et al.^2^. Shah et al.^3^ studied flow of hybrid nanoliquid under the influence of magnetic field. Anwar et al.^4^ addressed characteristics of shape factor for heat transfer in hybrid nanolqiuid. Melting effect on bio-convectional nanoliquid flow with second order slip has been analyzed by Waqas et al.^5^. Shah et al.^6^ reported thermophoresis and brownian motion effects on CNTs flow. Aspects of Casson nanomaterial on Darcy-Forhheimer flow is studied by Islam et al.^7^. Optimal thermal characteristics of MHD nanoliquid flow in variable thickness channel has been documented by Zeeshan et al.^8^. Zeeshan et al.^9^ has analyzed heat and mass transfer of MHD nanoliquid flow under the influences of radiation, viscous dissipation and Joule heating. Further studies of nanofluids are cited therein^10–12^. Khan et al.^13^ addressed MHD nanoliquid with entropy generation in rotating frame. Sohail et al.^14^ investigated Darcy Forchheimer hybrid nanoliquid in porous medium under the impact of entropy analysis. Khan et al.^15^ analyzed non-axisymmetric Homann stagnation point nanofluid flow through multiple solutions. Huda et al.^16^ elaborated Cattaneo–Christov model for nanofluid with moving needle. Reactive stretched flow of $\mathrm{Al}_{2}\mathrm{O}_{3}-water$ in porous space is examined by Lia et al.^17^.

Magnetic field application in fluid flow analysis has gained attention of scientists due to its wide range of utilization in many fields namely industries, drug delivery, MHD (Magnetohydrodynamics) generator, mechanical and physiological phenomena and many others. Nadeem et al.^18^ studied MHD nanoliquid flow numerically. Malvandi et al.^19^ studied mixed convection of MHD nanofluid saturate in vertical annulus. Activation energy in MHD squeezed flow with binary chemical reactions was studied by Ahmad et al.^20^. Hayat et al.^21^ investigated magnetohydrodynamics third grade nanoliquid convective flow by nonlinear stretched plate. Partial slip in MHD nanoliquid flow with viscous dissipation near stagnation point was investigated by Emad et al.^22^.

Radiation does not need any medium to transmit. It depends on shape, temperature and propagates by electromagnetic waves. It is practiced that system in industries having little temperature difference in fluid caused problems. To overcome this difficulty the researchers incorporated a term named as radiation parameter. The variation in temperature of fluid and wall can be novel by this parameter. Cortell^23^ summarized influence of heat generation and radiation in convective flow. Radiative MHD nanoliquid flow with convective condition has been discussed by Nadeem et al.^24^. Mohammadein et al.^25^ studied influence of thermal radiation on MHD nanoliquid flow with suction/injection. Effect of heat source and radiation on MHD CNTs liquid flow in rotating frame is studied by Muhammad et al.^26^. Hayat et al.^27^ explored non-Darcy flow of CNTs liquid subject to radiation and heat source.

Chemical reactions are categorized mainly in two types namely homogeneous and heterogeneous reactions. Reactions which encounter catalyst in same phase (namely gases, liquids, solids) correspond to homogeneous and reactions which happen in two or several different phases (like solid and gas, solid and liquid) as heterogeneous reactions. Some utilization of chemical reactions are found in iron oxidation, polymer and metallurgical industries. Reactions species have composite link for formation and usage of reactant species. Generally reactions rate depend on the magnitude of mass itself. A simple isothermal model proposed by Merkin et al.^28^ investigates homogeneous-heterogeneous reactions in flow. Influence of chemical reaction in liquid flow was studied by Bhattacharyya^29^. Chemical reactive fluid flow was reported by Rashidi et al.^30^ to explore mixed convection for heat and mass transfer. Convective flow with homogeneous-heterogeneous reactions saturated a porous medium was analyzed by Hayat et al.^31^. Zakir et al.^32^ studied CNTs in flow of liquid by stretched cylinder with Darcy–Forhheimer effect.

This work aims to address the analysis specifically in six dimensions. First of these is the formulation of problem, computation and then associated analysis. Secondly the comparative analysis of CNTs liquid under the influence of applied magnetic field. An induced magnetic field has been neglected for low Reynolds number. Third to study the effects of temperature dependent viscosity, viscous dissipation and Joule heating. Fourthly to examine the mixed convection and magnetohydrodynamics on CNTs liquid flow. Fifth heat transfer has been explored via thermal radiation and heat source/sink. Sixth mechanism of homogeneous and heterogeneous reactions are disclosed.

The current study contributes mathematical modeling, computation and comparison of associated analysis. To the best of authors’ knowledge, no one has attempted comparative study of SWCNTs-liquid and MWCNTs-liquid under the influences of magnetohydrodynamics, mixed convection, temperature dependent viscosity, thermal radiation, heat source sink/sink, viscous dissipation and Joule heating and homogeneous–heterogeneous reactions. This analysis is a new contribution in this dimension.

Water is treated as a base fluid for the submerging particles namely multi walled and single walled nanotubes. Water and nanomaterials are considered thermally balanced. The problem formulation has been carried out in cylindrical coordinates. System of nonlinear differential equations are changed to nonlinear ordinary differential equation via suitable transformations. Effects of various variables on velocity, temperature, skin friction and Nusselt number have been studied through graphical and tabulated outcomes.

CNTs namely SWCNTs and MWCNTs are seamless cylinders containing at least one layer of graphene (SWCNTs or MWCNTs) having closed or open ends. CVD (chemical vapor deposition) is the strong production of high CNTs volume mode that normally utilizes fluidized bed-reactors which strengthen diffusion of uniform gases and heat transport to metal catalyst nanomaterials. For this understanding, CNTs nanoliquid flow with varying viscosity of base liquid is one of the interesting discussion under consideration.

## Problem development

**Figure 1:**
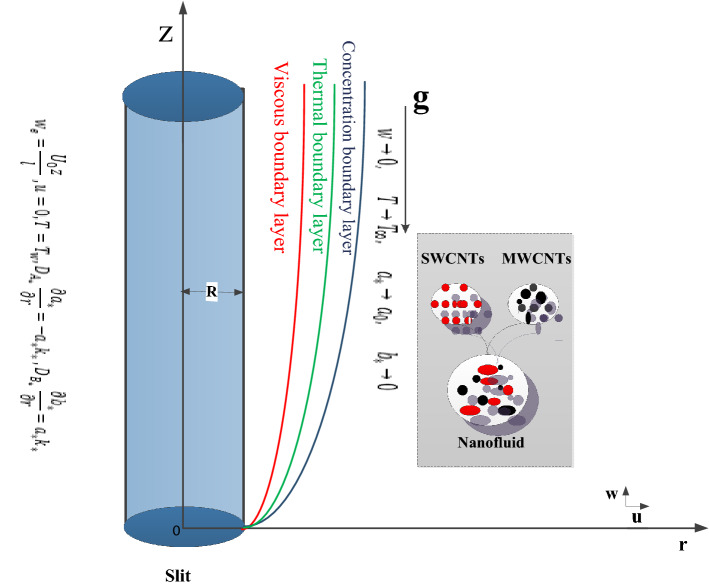
Schematic representation of problem.

In this analysis, it is considered that two-dimensional incompressible mixed convective flow of CNTs nanoliquid by stretchable cylinder. The liquid flow is caused by stretching cylinder. The viscosity of base liquid varies with the variation of temperature. Base liquid contains homogeneous combination of CNTs particles. Further more, CNTs particles and base liquid are in thermal equilibrium. Linearly stretching cylinder $(i.e.\quad w_e = \frac{U_0z}{l})$ is along axial direction $(z-axis)$ while liquid is assumed to deform in radial direction $(r-axis)$. Figure 1 addresses the geometric configuration of flow problem. Heat transfer characteristics are explored via heat generation/absorption, viscous dissipation and Joule heating. Diffusion species are accounted in base liquid for reactions.

The fluid viscosity^33^ is treated inversely linear function of temperature i.e.,

1 $$\begin{aligned} {\left\{ \begin{array}{ll} &{}\frac{1}{\mu (T)} = \frac{1}{\mu _\infty }\left( 1 + \gamma _0(T-T_\infty )\right) = \frac{\gamma _0}{\mu _\infty }\left( T-T_r \right) = a_{1}\left( T-T_r\right) = \frac{1}{\mu _\infty }\left( \frac{\theta _r-\theta }{\theta _{r}}\right) ,\\ &{}\mu (T) = \mu _\infty \left( \frac{\theta _{r}}{\theta _{r}-\theta }\right) , \end{array}\right. } \end{aligned}$$

here

$$\begin{aligned} {\left\{ \begin{array}{ll} T_{r} = \left( T_{\infty } -\frac{1}{\gamma _{0}}\right) , \gamma _{0} = \left( \frac{1}{T_{\infty } - T_{r}}\right) , \theta _{r} = \left( \frac{T_{r}-T_{\infty }}{T_{w}-T_{\infty }}\right) = \left( \frac{1}{\gamma _{0}(T_{w}-T_{\infty })}\right) , a_{1} = \left( \frac{\gamma _{0}}{\mu _{\infty }}\right) , \end{array}\right. } \end{aligned}$$

where the constants $\left( T_{r}\right)$ and $\left( a_{1}\right)$ depend on the initial state and thermal characteristics $\left( \gamma _{0}\right)$ of the liquid. It is often scaled that $\left( a_{1}>0\right)$ and $\left( a_{1}< 0\right)$ for liquids and gases respectively. The values of $\left( \theta _{r}\right)$ is computed by viscosity of liquid under consideration and the difference of operating temperature. Larger $\left( \theta _{r}\right)$ corresponds to smaller $\left( \gamma _{0}\right)$ or $\left( T_{w}-T_{\infty }\right)$. Thus the influence of varying viscosity can be ignored while the smaller $\left( \theta _{r}\right)$ tends to either the liquid viscosity changes remarkably or high difference of operating temperature. The varying viscosity changes to constant $\left( \mu = \mu _{\infty }\right)$ for $\theta _{r}->\infty$ $(i.e. \ \gamma _{0}->0)$.

For heterogeneous-homogeneous reactions, the model of isothermal^28^ is defined by

2 $$\begin{aligned} \mathbf {A}_{\star }+2\mathbf {B}_{\star }\rightarrow 3\mathbf {B}_{\star },rate=k_{p}a_{\star }b_{\star }^{2}. \end{aligned}$$

At surface of catalyst the first-order reaction is

3 $$\begin{aligned} \mathbf {A_{\star }}\rightarrow \mathbf {B_{\star }},rate=k_{\star }a_{\star }. \end{aligned}$$

Where ($a_{\star }$, $b_{\star }$) stand for concentrations of species ($\mathbf {A}_{\star }$, $\mathbf {B}_{\star }$), ($k_{p}$, $k_{\star }$) show the rate constants. The auto-catalyst ($\mathbf {B}_{\star }$) is taken inside the boundary layer and outside it is dealt equal concentration ($a_{0}$) of reactant ($\mathbf {A_{\star }}$). Equation (3) reveals that there is no reaction rate outside the boundary layer.

The governing problems via boundary layer approximation in cylindrical coordinates are as follows^28,34^:

4 $$\begin{aligned} \frac{\partial \left( ru\right) }{\partial r}+\frac{\partial \left( rw\right) }{\partial z}=0, \end{aligned}$$

5 $$\begin{aligned} w\frac{\partial w}{\partial z}+u\frac{\partial w}{\partial r}&=\frac{1}{\rho _{nf}} \left( \frac{\partial \mu _{nf}(T)}{\partial r}\frac{\partial w}{\partial r}+ \frac{1}{r}\mu _{nf}(T)\frac{\partial w}{\partial r}+\mu _{nf}(T)\frac{\partial ^{2}w}{\partial r^{2}}\right) \nonumber \\&\quad +gB(T-T_{\infty }) - \frac{\sigma _{nf}}{\rho _{nf}}B_{0}^{2}w, \end{aligned}$$

6 $$\begin{aligned} u\frac{\partial T}{\partial r}+w\frac{\partial T}{\partial z}&=\frac{k_{nf}}{(C_{p}\rho )_{nf}}\left( \frac{\partial ^{2}T}{\partial r^{2}}+ \frac{1}{r}\frac{\partial T}{\partial r}\right) + \frac{16 \sigma ^{\star } T_{0}^{3}}{3k^{\star } (C_{p}\rho )_{nf}}\left( \frac{\partial ^{2} T}{\partial r^{2}}+ \frac{1}{r}\frac{\partial T}{\partial r}\right) \nonumber \\&\quad + \frac{\mu _{nf}(T)}{(C_{p}\rho )_{nf}}\left( \frac{\partial w}{\partial r}\right) ^{2} + \frac{Q_{0} (T-T_{\infty })}{(C_{p}\rho )_{nf}}, \end{aligned}$$

7 $$\begin{aligned} u\frac{\partial a_{\star }}{\partial r}+w\frac{\partial a_{\star }}{\partial z}&=D_{A_{\star }}\left( \frac{\partial ^{2}a_{\star }}{\partial r^{2}}+\frac{\star }{r}\frac{\partial a_{\star }}{\partial r }\right) -k_{p}a_{\star }b_{\star }^{2}, \nonumber \\ u\frac{\partial b_{\star }}{\partial r}+w\frac{\partial b_{\star }}{\partial z}&=D_{B_{\star }}\left( \frac{\partial ^{2}b_{\star }}{\partial r^{2}}+\frac{1}{r}\frac{\partial b_{\star }}{\partial r }\right) +k_{p}a_{\star }b_{\star }^{2}, \end{aligned}$$

with conditions

8 $$\begin{aligned} {\left\{ \begin{array}{ll} &{}w_{e}=\frac{U_{0}z}{l},\quad u=0, \quad \quad T = T_{w},\\ &{}\quad D_{A_{\star }}\frac{\partial a_{\star }}{\partial r}= - a_{\star }k_{\star },\quad D_{B_{\star }}\frac{\partial b_{\star }}{\partial r}= a_{\star }k_{\star }\quad at \quad r=R,\\ &{}w\rightarrow 0,\quad T\rightarrow T_{\infty },\quad a_{\star }\rightarrow a_{0},\quad b_{\star }\rightarrow 0 \quad as \quad r\rightarrow \infty , \end{array}\right. } \end{aligned}$$

where (*w*, *u*) the velocity components, $(\rho _{nf})$ the density of nanoliquid, $((C_{p})_{nf})$ the specific heat of nanoliquid, $(\mu _{nf} (T))$ the dynamic viscosity of nanomaterial, $(k_{nf})$ the thermal conductivity nanoliquid, $(w_{e})$ the stretching velocity, $(U_{0})$ the reference velocity, (*l*) the characteristics length, (*R*) the radius of cylinder, $(B_{0})$ the strength of magnetic field, $((\sigma )_{nf})$ electric transport of nanofluid, (*g*) the gravitational acceleration, (*B*) the thermal coefficient, $(Q_{0}(T-T_\infty ))$ the heat generate per unit volume, ($T_{w}$, $T_{\infty }$) the wall and ambient temperatures, $(\sigma ^{\star })$ the Stefan Boltzmann constant, $(k^{\star })$ the mean absorption coefficient and $(D_{A_{{\star }}}$, $D_{B_{{\star }}})$ the diffusion coefficients ($\mathbf {A_{\star }}$, $\mathbf {B_{\star }}$) respectively.

Following models^35–37^ one has

9 $$\begin{aligned} \mu _{nf} =&\frac{\mu _{f}(T)}{\left( 1-\phi \right) ^{2.5}},\quad \rho _{nf}=\left( 1-\phi \right) \rho _{f}+\phi \left( \rho \right) _{CNT},\nonumber \\ \left( C_{p}\rho \right) _{nf} =&\left( 1-\phi \right) \left( C_{p}\rho \right) _{f}+\phi \left( C_{p}\rho \right) _{CNT}, \nonumber \\ \frac{k_{nf}}{k_{f}}=&\frac{\left( 1-\phi \right) +2\phi \frac{k_{CNT}}{k_{CNT}-k_{f}}\ln \frac{k_{CNT}+k_{f}}{2k_{f}}}{\left( 1-\phi \right) +2\phi \frac{kf}{k_{CNT}-k_{f}}\ln \frac{k_{CNT}+k_{f}}{2k_{f}}}, \nonumber \\ \frac{\sigma _{nf}}{\sigma _{f}} =&\frac{3 (\sigma -1) \phi }{(\sigma + 2)- (\sigma -1)\phi } + 1, \quad \sigma = \frac{\sigma _{CNT}}{\sigma _{f}}, \end{aligned}$$

in which $\left( \phi \right)$ denotes the volume fraction of nanosized particle, $\left( \rho \right) _{f}$ the base liquid density, $\left( \mu \right) _{f}$ the dynamic viscosity of base liquid, $\left( \rho \right) _{CNT}$ the density of nanotubes, $\left( k\right) _{f}$ the base liquid conductivity, $\left( k\right) _{CNT}$ represents nanotubes thermal conductivity, $\left( \sigma \right) _{CNT}$ and $\left( \sigma \right) _{f}$ the electric conductivity of base liquid and nanoparticle respectively.

**Table 1:** Thermophysical characteristics of CNTs liquid^10,36^.

Physical properties	Base fluid	Nanoparticles	
	Water	SWCNTs	MWCNTs
$\rho \, (\mathrm{kg}/\mathrm{m}^{3})$	997	2600	1600
$C_{p}\,(\mathrm{J}/\mathrm{kg K})$	4179	425	796
$k\,(\mathrm{W}/\mathrm{mK})$	0.613	6600	3000
$\sigma \,(\mathrm{S}/\mathrm{m})$	0.005	$1\times 10^{7}$	$1\times 10^{7}$
*Pr*	6.2	–	–

Where $(\rho , C_{p}, k, \sigma )$ the density, specific heat, thermal conductivity and electric conductivity respectively.

Letting

10 $$\begin{aligned} \eta =&\sqrt{\frac{U_{0}}{(\nu _{\infty })_{f} l}}\left( \frac{r^{2}-R^{2}}{2R}\right) ,\quad \psi =\sqrt{w_{e}(\nu _{\infty })_{f} z}Rf\left( \eta \right) \quad w =\frac{U_{0}z}{l}f^{\prime }\left( \eta \right) , \nonumber \\ u=&-\sqrt{\frac{(\nu _{\infty })_{f} U_{0}}{l}}\frac{R}{r}f\left( \eta \right) ,\quad \theta \left( \eta \right) =\frac{T-T_{\infty }}{T_{w}-T_{\infty }},\quad \Phi _{\star } (\eta )=\frac{a_{\star }}{a_{0}},\quad h_{\star }(\eta )=\frac{b_{\star }}{a_{0}}. \end{aligned}$$

Equation (4) is trivially confirmed and Eqs. (5 – 8) reduce to

11 $$\begin{aligned}&\left( 1+2\gamma \eta \right) f^{\prime \prime \prime }+2 \gamma f^{\prime \prime } - \left( \frac{1}{\theta _r-\theta }\right) \left( 1+2\gamma \eta \right) \theta ^{\prime }f^{\prime \prime }\nonumber \\&\quad + \left( \frac{\theta _r-\theta }{\theta _{r}}\right) \left( 1-\phi \right) ^{2.5}\left( 1-\phi +\phi \frac{(\rho )_{CNT}}{(\rho )_{f}}\right) \left( ff^{\prime \prime } -(f^{\prime })^{2}\right) \nonumber \\&\quad +\left( \frac{\theta _r-\theta }{\theta _{r}}\right) \left( 1-\phi \right) ^{2.5}\left( 1-\phi +\phi \frac{(C_{p}\rho )_{CNT}}{(C_{p}\rho )_{f}}\right) Gr\theta \nonumber \\&\quad -\left( \frac{\theta _r-\theta }{\theta _{r}}\right) \left( 1-\phi \right) ^{2.5}\left( \frac{3 (\sigma -1) \phi }{(\sigma + 2)- (\sigma -1)\phi } + 1\right) M_{f}f^{\prime } = 0, \end{aligned}$$

12 $$\begin{aligned}&\left( \frac{(k)_{nf}}{(k)_{f}}+ \frac{4}{3}R_{d}\right) \left( (1 + 2\gamma \eta )\theta ^{\prime \prime } + 2\gamma \theta ^{\prime }\right) + Pr\left( Q_{r}\theta + \frac{(C_{p}\rho )_{nf}}{(C_{p}\rho )_{f}}f\theta ^{\prime }\right) \nonumber \\&\quad + Pr\left( 1-\phi \right) ^{2.5}\left( \frac{\theta _r}{\theta _r-\theta }\right) Ec(1 + 2\gamma \eta )(f^{\prime \prime })^{2}= 0, \end{aligned}$$

13 $$\begin{aligned}&\frac{1}{Sc}\left( (1+2\gamma \eta )\Phi _{\star }^{\prime \prime }+\gamma \Phi _{\star }^{\prime }\right) +f \Phi _{\star }^{\prime }-K\Phi _{\star } h_{\star } ^{2}=0, \end{aligned}$$

14 $$\begin{aligned}&\frac{\delta _{\star } }{Sc}\left( (1+2\gamma \eta )h_{\star } ^{\prime \prime }+\gamma h_{\star }^{\prime }\right) +f h_{\star } ^{\prime }+K \zeta _{\star } h_{\star }^{2}=0, \end{aligned}$$

with

15 $$\begin{aligned}&f(0)=0,\quad f^{\prime }(0)=1,\quad \theta (0)= 1, \quad \Phi _{\star } ^{\prime }(0)= Ks\Phi _{\star } (0), \quad \delta _{\star } h ^{\prime }(0)=-Ks\Phi _{\star } (0), \nonumber \\&f^{\prime }(\eta ) = 0, \quad \theta \left( \eta \right) =0, , \quad \Phi _{\star } (\eta )\rightarrow 1,\quad h_{\star } (\eta )\rightarrow 0, \quad as \quad \eta \rightarrow \infty , \end{aligned}$$

where $\left( \gamma =\frac{1}{R}\sqrt{\frac{(\nu _\infty )_{f} l}{U_{0}}}\right)$ depicts the curvature parameter, $\left( Gr = \frac{\lambda _{1}}{Re_z^{2}}\right)$ the mixed convection variable, $\left( Re_{z} = \frac{w_{e}z}{(\nu _{\infty })_{f}}\right)$ the local Reynolds number, $\left( \lambda _{1} = \frac{g(T_{w}-T_{\infty })Bz^{3}}{(\nu _{\infty })_{f}^{2}}\right)$ local Grashof number, $\left( M_{f} = \frac{\beta _{0}^{2}\sigma _{f} l}{(\rho _{\infty })_{f} U_{0}}\right)$ the Hartman number, $\left( Ec = \frac{w_{e}^{2}}{(C_{p})_{f} (T_{\infty }-T_{w})}\right)$ the Eckert number, $\left( R_{d} = \frac{4 \sigma ^{\star }T_{0}^{3}}{3 k^{\star }k_{f}}\right)$ the radiation parameter, $\left( Q_{r}= \frac{Q_{0}l }{U_{0}(\rho _{\infty } C_{p})_{f}}\right)$ the heat generation/absorption parameter, $\left( K=\frac{k_{p}a_{0}^{2}l}{U_{0}}\right)$ the homogeneous variable, $\left( Ks=\frac{k_{\star }}{D_{A_{\star }}}\sqrt{\frac{(\nu _{\infty })_{f} l}{U_{0}}}\right)$ the heterogeneous variable, $\left( Sc= \frac{(\nu _\infty )_{f} }{D_{A_{\star }}}\right)$ the Schmidt number, ${\text{Pr}} =\frac{ \left({\mu_\infty \, C_{p}}\right)_{f} }{ k_f}$ and $\left( \delta _{\star }=\frac{D_{B_{\star }}}{D_{A_{\star }}}\right)$ the diffusion ratio coefficient. The Prandtl and Schmidt numbers have been kept constant throughout the study.

One has, for equal diffusion coefficients $D_{A_{\star }}$ and $D_{B_{\star }}$ as^28^;

$$\begin{aligned} \Phi _{\star }(\eta )+h_{\star }(\eta )=1. \end{aligned}$$

Equations (13)–(14) imply that

16 $$\begin{aligned}&(1+2\gamma \eta ) \Phi _{\star }^{\prime \prime }+ 2\gamma \Phi _{\star } ^{\prime }+Scf \Phi _{\star }^{\prime }-ScK \Phi _{\star } (1- \Phi _{\star } )^{2}=0. \end{aligned}$$

17 $$\begin{aligned}&\Phi _{\star } ^{\prime }(0)= Ks \Phi _{\star } (0), \quad \Phi _{\star } (\eta )\rightarrow 1 \quad as \quad \eta \rightarrow \infty . \end{aligned}$$

Skin friction coefficient and Nusselt number are

18 $$\begin{aligned} C_{f}=\frac{\tau _{w}}{\frac{\rho U_{0}^{2}z^{2}}{2l^{2}}},\quad \quad Nu_{z}=\frac{zq_{w}}{k_{f}(T_{w}-T_{\infty })}, \end{aligned}$$

where $\tau _{w}$ and $q_{w}$ are defined as;

19 $$\begin{aligned} {\left\{ \begin{array}{ll} &{}\tau _{w}=-\mu _{f} \left( \frac{\partial w}{\partial r}\right) _{r=R},\quad \quad q_{w}=-\left( k_{nf}+\frac{16\sigma ^{\star }T_{0}^{3}}{3k^{\star }}\right) \left( \frac{\partial T}{\partial r}\right) _{r=R},\\ &{}C_{f}{Re}_{z}^{\frac{1}{2}}=-\frac{1}{(1-\phi )^{2.5}}f^{\prime \prime }(0) ,\quad \quad Nu_{z}{Re}_{z}^{-\frac{1}{2}}=-\left( \frac{k_{nf}}{k_{f}}+\frac{4}{3}R_{d}\right) \theta ^{\prime }\left( 0\right) , \end{array}\right. } \end{aligned}$$

where ${Re}_{z}^{\frac{1}{2}}=\sqrt{\frac{U_{0}}{(\nu _\infty )_{f}l}}z$ represents the local Reynolds number.

## Discussion

This section elaborates the graphical discussion for SWCNTs and MWCNTs nanofluids. SWCNTs is indicated by the solid lines while MWCNTs by dashed lines. The values Curvature variable $\left( \gamma \right)$ = Hartman number $\left( M_{f}\right)$ = Mixed convection $\left( Gr\right)$ = Volume fraction $\left( \phi \right)$ = Radiation parameter $\left( R_{d}\right)$ = Variable viscosity parameter $\left( \theta _{r}\right)$ = Eckert number $\left( Ec\right)$ = 0.1, Prandtl number $\left( Pr = 6.2\right)$, Homogeneous reaction variable $\left( K = 0.7\right)$, Heterogeneous reaction variable $\left( Ks = 0.9\right)$ and Schmidt number $\left( Sc = 1.2\right)$ are taken for outcomes. Further the values of variables are taken constant except the specific variable considered in Figures. The thermophysical characteristics of CNTs nanoliquid is defined in Table 1. In Table 1, $(\rho , C_{p}, k)$^10^ denote the density, the specific heat and the thermal conductivity while $(\sigma )$^36^ represents the electric conductivity. The outcomes of curvature, Hartman number, Grashof number, volume fraction and others involved dimensionless variables are elaborated for the distributions, velocity $\left( f^{\prime }(\eta )\right)$, temperature $\left( \theta (\eta )\right)$, concentration $\left( \Phi (\eta )\right)$, Skin friction coefficient $\left( C_{f}Re_{z}^{\frac{1}{2}}\right)$, Nusselt number $\left( Nu_{z}Re_{z}^{\frac{-1}{2}}\right)$.

### Velocity profile

**Figure 2:**
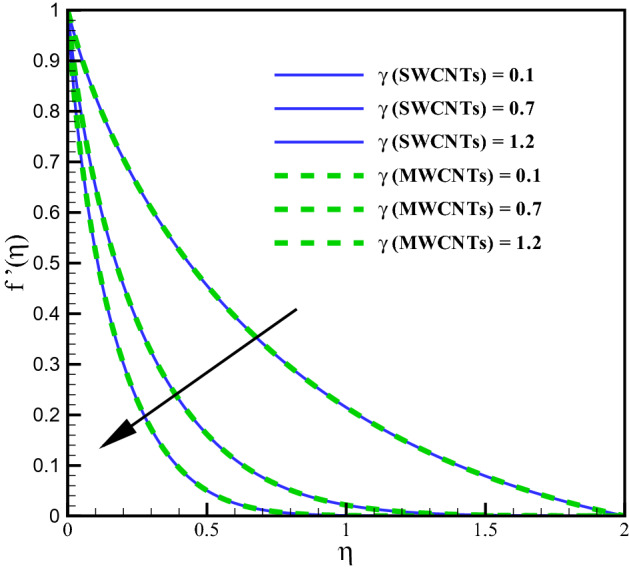
Curves via $\gamma$ for $f^{\prime }(\eta )$.

**Figure 3:**
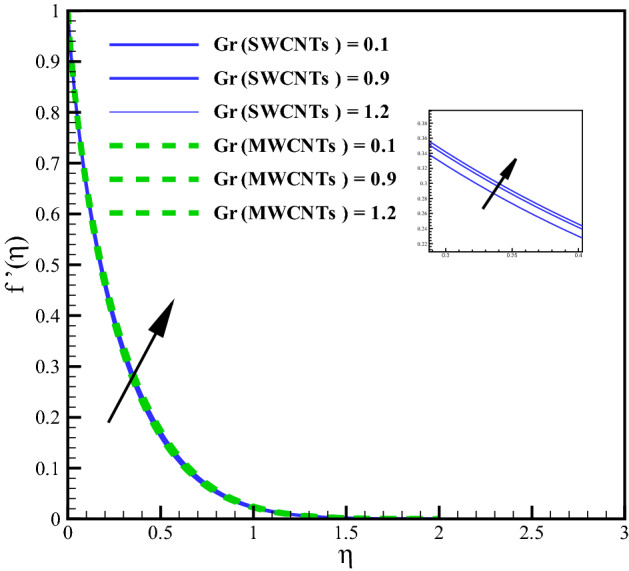
Curves via *Gr* for $f^{\prime }(\eta )$.

**Figure 4:**
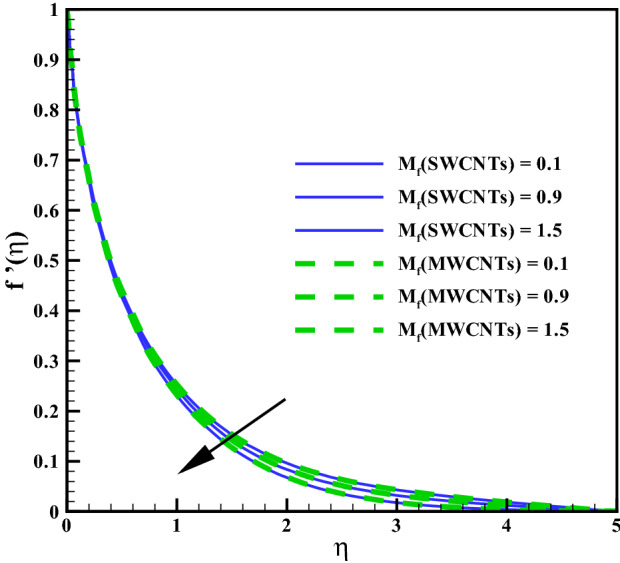
Curves via $M_{f}$ for $f^{\prime }(\eta )$.

**Figure 5:**
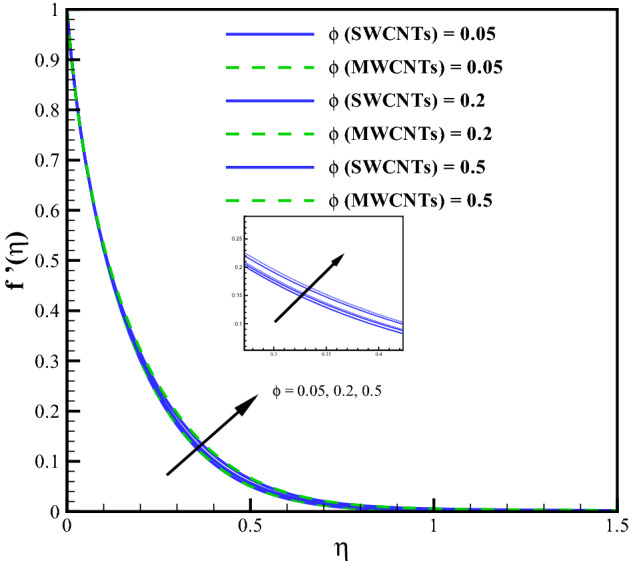
Curves via $\phi$ for $f^{\prime }(\eta )$.

Larger curvature variable $(\gamma )$ declines the velocity distribution (see Fig. 2). Clearly $(\gamma )$ and (*R*) inversely relate to each other and therefore, the resistive force enhances for the nanoliquid flow. The velocity profile thus declines. Figure 3 is sketched to discuss the effect of mixed convection variable (*Gr*) on velocity field. From the figure, it is observed that velocity enhances for both SWCNTs and MWCNTs. Because the mixed convection variable directly relates to thermal buoyancy forces and the resistive force becomes less against larger (*Gr*). Consequently, the velocity profile increases. Figure 4 shows curves of Hartman number $(M_{f})$ for velocity. The flow field declines for larger $(M_{f})$. Lorentz force rises for higher $(M_{f})$ and thus the velocity of liquid decreases. The velocity fields for SWCNTs and MWCNTs behaves similarly against $(\gamma )$, (*Gr*) and $(M_{f})$. Impact of volume fraction for SWCTs-water and MWCNTs-water is addressed in Fig. 5. Nanoliquid flow boosts when the values of $(\phi )$ increase. Physically, larger $(\phi )$ caused more convective flow that enhances the liquid velocity. Furthermore, the flow by submerging MWCNTs nanomaterial is observed higher than by adding SWCNTs nanoparticles. Because the density of MWCNTs is lighter when compared to SWCNTs density.

### Temperature profile

**Figure 6:**
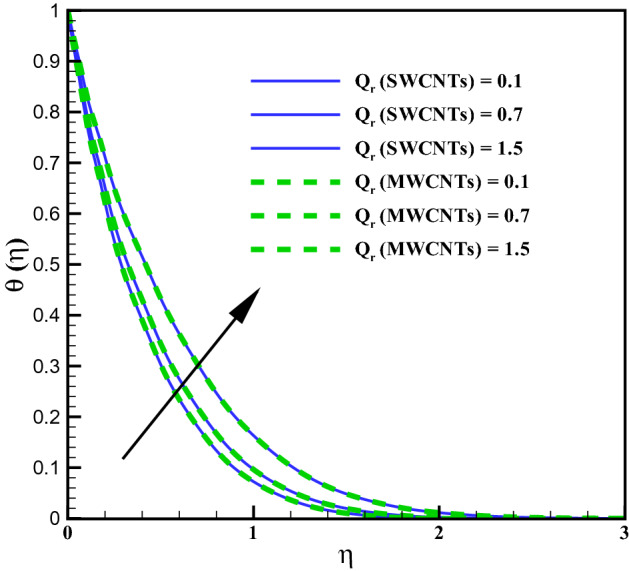
Curves via $Q_r$ for $\theta (\eta )$.

**Figure 7:**
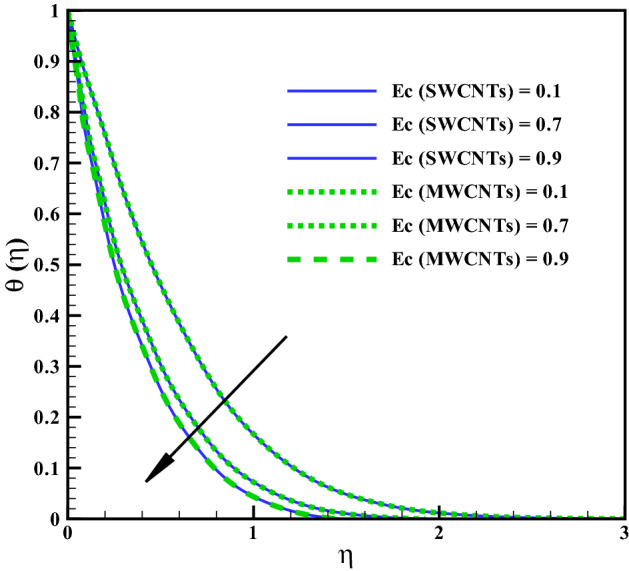
Curves via Ec for $\theta (\eta )$.

**Figure 8:**
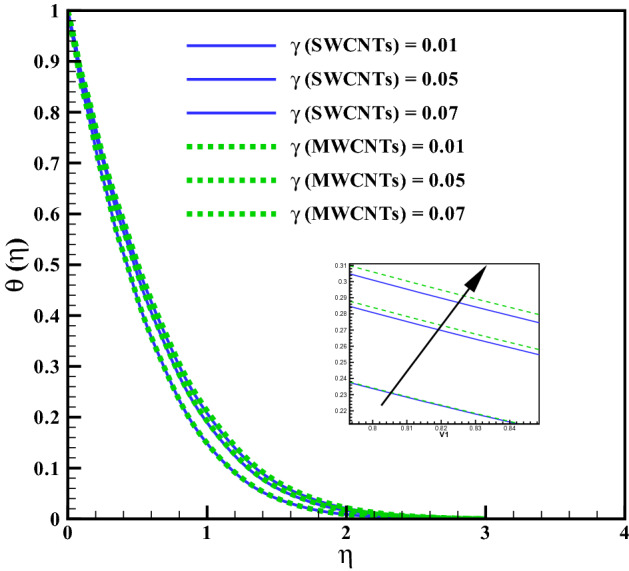
Curves via $\gamma$ for $\theta (\eta )$.

**Figure 9:**
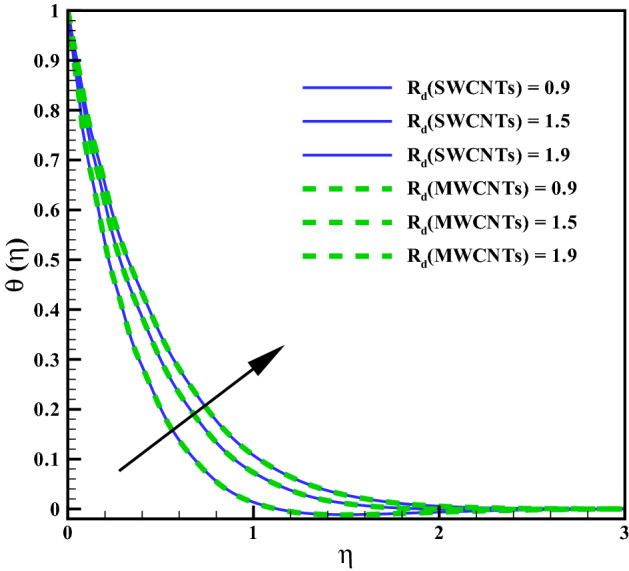
Curves via $R_{d}$ for $\theta (\eta )$.

**Figure 10:**
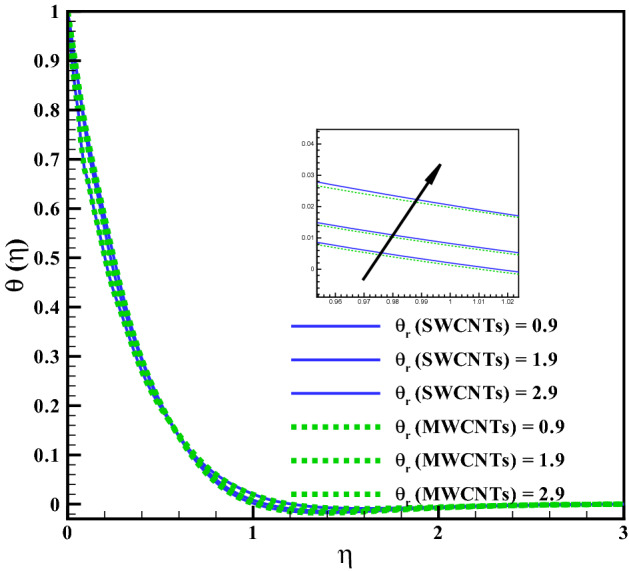
Curves via $\theta _{r}$ for $\theta (\eta )$.

The curves for temperature against heat generation variable $(Q_{r})$ has been presented in Fig. 6. Thermal layer enhances via larger $(Q_{r})$. Heat transfer increases against larger $(Q_{r})$ due to the direct relation with thermal constant coefficient. Temperature variations is noted similar for both type CNTs. The temperature curves follow free stream condition for larger $\eta$. Figure 7 addresses the influence of viscous dissipation (i.e. Eckert number (*Ec*)) on temperature. The temperature rises when (*Ec*) is increased. Larger *Ec* leads to higher the kinetic energy of liquid molecules resulting more collision between liquid molecules. Consequently heat produce, due to collisions and the temperature rises significantly. Moreover, same temperature behavior is noted for cylinder shaped SWCNTs and MWCNTs nanofluids. Figure 8 shows the effect of curvature variable on temperature. The fluid heats up by increasing $(\gamma )$. Higher values of curvature variable reduce the radius of stretchable cylinder. Subsequently heat produce due to friction that enhances the temperature for the nanoliquid. Moreover, the temperature of liquid is noted higher by addition of MWCNTs nanomaterials than SWCNTs nanomaterials. The curves of radiation variable $(R_{d})$ for $(\theta (\eta ))$ is shown in Fig. 9. Outcomes of radiation variable results enhancement in temperature $(\theta (\eta ))$ . Higher values of radiation variable results increase in heat flux at the surface. Hence, the thermal layer thickness increases. Figure 10 is sketched for the temperature dependent viscosity variable on temperature field. Figure shows that increment in $(\theta _{r})$, contributes first declines and than enhances the temperature. Physically, it reflects that smaller $(\theta _{r})$ tends to remarkable change in viscosity or the higher the difference of operating temperature. However, opposite trend is noted for larger $(\theta _{r})$. Hence the temperature first declines and than enhances. The temperature is noted higher for SWCNTs near the surface than MWCNTS while the temperature is observed higher far away from the surface when compared with SWCNTs.

### Concentration profile

**Figure 11:**
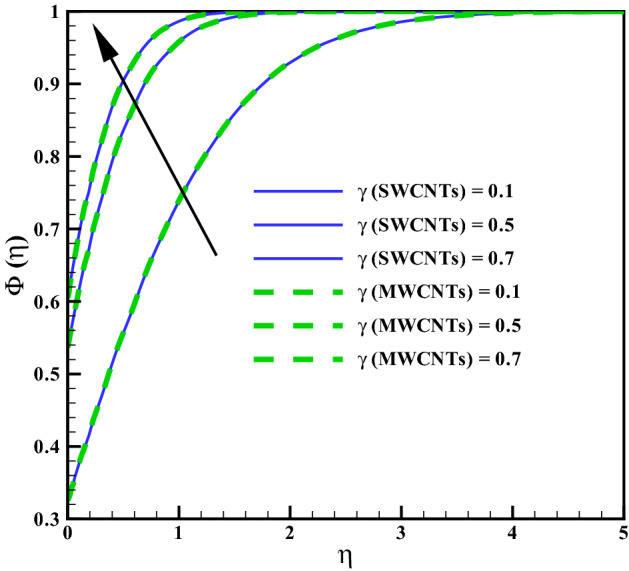
Curves via $\gamma$ for $\Phi (\eta )$.

**Figure 12:**
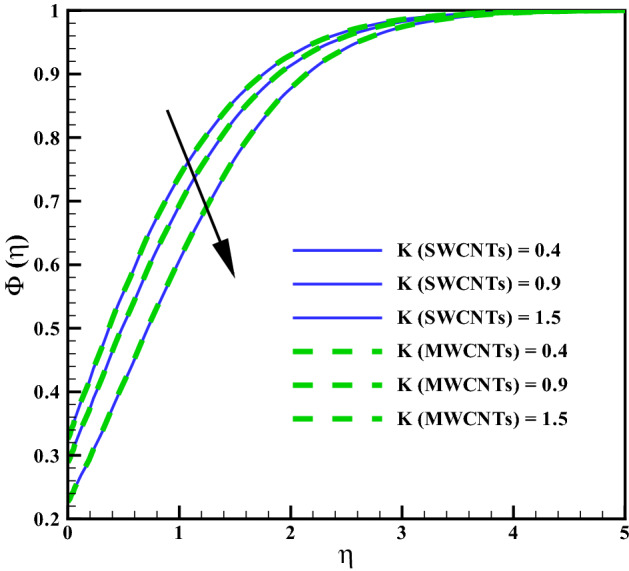
Curves via *K* for $\Phi (\eta )$.

**Figure 13:**
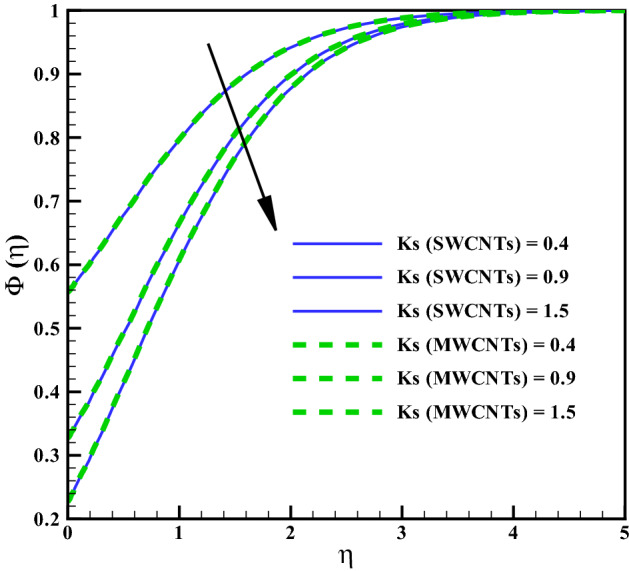
Curves via *Ks* for $\Phi (\eta )$.

**Figure 14:**
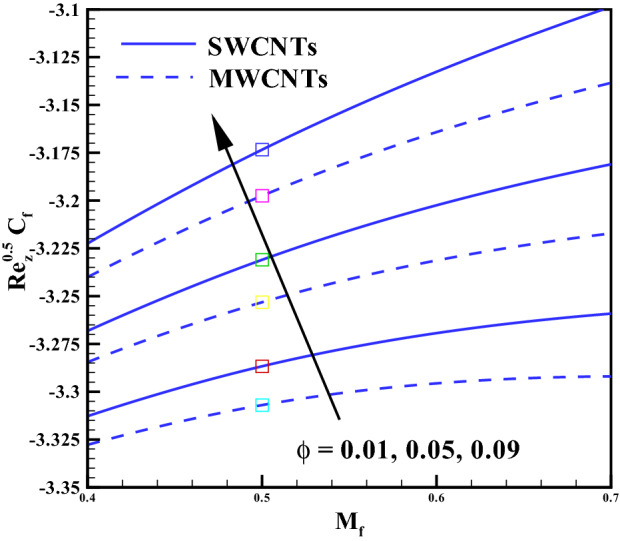
Plots for $C_{f}Re^{\frac{1}{2}}_{z}$ via $M_{f}$ and $\phi$.

**Figure 15:**
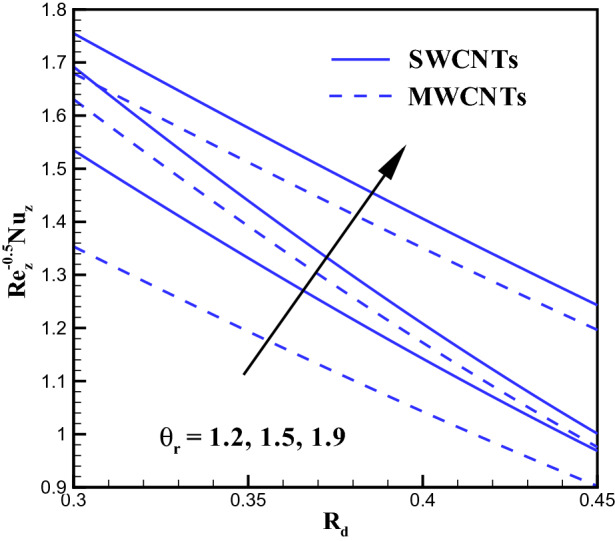
Plots for $Nu_{z}Re^{-0.5}_{z}$ via $R_{d}$ and $\theta _{r}$.

**Figure 16:**
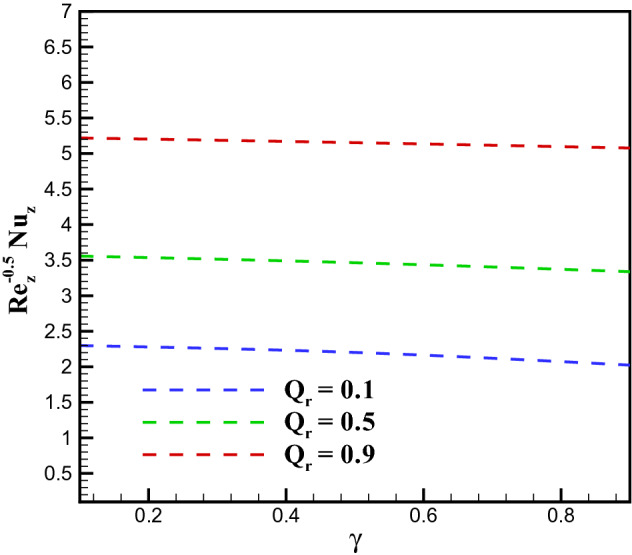
Plots for $Nu_{z}Re^{-0.5}_{z}$ via $\gamma$ and $Q_{r}$.

Figure 11 addresses the concentration profile via larger curvature variable $(\gamma )$. The Concentration profile enhances against $(\gamma )$. Physically, the resistive forces produce between liquid molecules for larger $(\gamma )$ due to inverse relation with radius of cylinder. Figure 12 shows the curves for concentration gradient via homogeneous variable (*K*). The solutat layer thickness increases for larger (*K*). In fact, there is direct relation between chemical reaction and the values of (*K*). The concentration for larger heterogeneous variable (*Ks*) can be seen in Fig. 13. Same behavior is noted against (*Ks*). Figure 14 shows the behavior of skin friction coefficients via $(M_{f})$ and $(\phi )$. Skin friction enhances for larges values of $(M_{f})$ and $(\phi )$. Resistive forces develop by addition of volume fraction and larger Hartman number. Figure 15 addresses the Nusselt number via variables $(R_{d})$ and $(Q_{r})$. Nusselt number increases for larger $Q_{r}$ and it decreases for $R_{d}$. Larger $(Q_{r})$ results small changes in viscosity or difference of operating temperature. Thus, heat transfer at the surface enhances. Nusselt number for $(\gamma )$ and $(Q_{r})$ is opposite (see Fig. 16). The skin friction can be controlled via $(M_{f})$ and $(\phi )$. Heat transfer rate at surface advances for larger $(Q_{r})$ and smaller $(R_{d})$ and $(\gamma )$.

## Main findings

After studying the governing problem via stretchable cylinder, the key results have been mentioned below:

1. The velocity fields decrease against curvature $(\gamma )$ and Hartman $(M_{f})$ variables and increase for larger volume fraction $(\phi )$ and mixed convection (*Gr*) variable. Furthermore same behavior is observed for SWCNTs and MWCNTs against Hartman, mixed convection and curvature variables. In addition, the velocity profile for MWCNTs nanofluid is noted higher than SWCNTs nanofluid against $(\phi )$.
2. Temperature is an increasing function of curvature variable $(\gamma )$ while for viscosity variable $(\theta _{r})$, the temperature decreases close to the surface and than increases far way from the surface. In addition, temperature in case of SWCNTs nanomaterials is observed higher when compared with MWCNTs nanomaterials against curvature and viscosity variables. Similar trend is observed for SWCNTs and MWCNTs against Eckert, Heat generation, mixed convection and Radiation variables.
3. The solutal layer thickness decreases against curvature variable $(\gamma )$ while opposite behavior has been seen against homogeneous-heterogeneous reactions variables (*K*, *Ks*).
4. Skin friction coefficient develops for $(M_{f})$ and it declines for larger $(\phi )$. Nusselt number $(Nu_{z}Re_{z}^{\frac{-1}{2}})$ boosts against $(\theta _{r})$ and it decreases via radiation and curvature variables.

The findings of current analysis have its usage in many phenomena like extrusion where the submerging particles to the liquid for cooling purpose under a certain temperature, wire drawing, condensation processes of metallic plate in bath and cooling glasses etc. It could help to develop catalytic phenomenon demanding the species reactions for the scientific community.
